# Incidence and Predictors of Clinically Significant Bleedings after Transcatheter Left Atrial Appendage Closure

**DOI:** 10.3390/ijerph192113802

**Published:** 2022-10-24

**Authors:** Kamil Zieliński, Radosław Pracoń, Marek Konka, Mariusz Kruk, Cezary Kępka, Piotr Trochimiuk, Mariusz Dębski, Edyta Kaczmarska, Jakub Przyłuski, Ilona Kowalik, Zofia Dzielińska, Andrzej Kurowski, Adam Witkowski, Marcin Demkow

**Affiliations:** 1 Department of Coronary and Structural Heart Diseases, National Institute of Cardiology, Alpejska 42, 04-628 Warsaw, Poland; 2 Department of Congenital Heart Diseases, National Institute of Cardiology, Alpejska 42, 04-628 Warsaw, Poland; 3 Clinical Research Support Center, National Institute of Cardiology, Alpejska 42, 04-628 Warsaw, Poland; 4 Department of Anesthesiology, National Institute of Cardiology, Alpejska 42, 04-628 Warsaw, Poland; 5 Department of Interventional Cardiology and Angiology, National Institute of Cardiology, Alpejska 42, 04-628 Warsaw, Poland

**Keywords:** left atrial appendage closure, bleeding, stroke, atrial fibrillation

## Abstract

Background: Transcatheter left atrial appendage closure (LAAC) is performed in patients unsuitable for long-term anticoagulation, predominantly due to prior bleeding events. The study aimed to investigate the incidence and predictors of clinically significant bleeding (CSB) post-LAAC. Methods: Consecutive patients after LAAC with an Amplatzer or WATCHMAN device were analyzed (05.2014–11.2019). Bleeding was classified as CSB when associated with at least one of the following: death, ≥2 g/dL hemoglobin drop, ≥2 blood units transfusion, critical anatomic site, or hospitalization/invasive procedure. Results: Among 195 patients (age 74 (68–80), 43.1% females, HAS-BLED score 2.0 (2.0–3.0)), during median follow-up of 370 (IQR, 358–392) days, there were 15 nonprocedural CSBs in 14 (7.2%) patients. Of those, 9 (60.0%) occurred during postprocedural dual antiplatelet therapy (DAPT) (median 46 (IQR: 16–60) days post-LAAC) vs. 6 (40%) after DAPT discontinuation (median 124 (81–210) days post-LAAC), translating into annualized CSB rates of 14.0% (per patient-year on DAPT) vs. 4.6% (per patient-year without DAPT). In 92.9% (13/14) of patients, the post-LAAC nonprocedural CSB was a recurrence from the same site as bleeding pre-LAAC. In the multivariable model, admission systolic blood pressure (SBP) > 127 mmHg (HR = 10.73, 1.37–84.26, *p* = 0.024), epistaxis history (HR = 5.84, 1.32–25.89, *p* = 0.020), permanent atrial fibrillation (AF) (HR = 4.55, 1.20–17.20, *p* = 0.025), and prior gastrointestinal bleeding (HR = 3.35, 1.01–11.08, *p* = 0.048) predicted post-LAAC CSB. Conclusions: Nonprocedural CSBs after LAAC, with a similar origin as the pre-LAAC bleedings, were observed predominantly during postprocedural DAPT and predicted by elevated admission SBP, prior epistaxis, permanent AF, and gastrointestinal bleeding history. Whether a more reserved post-LAAC antiplatelet regimen and stringent blood pressure control may improve LAAC outcomes remains to be studied.

## 1. Introduction

Transcatheter left atrial appendage closure (LAAC) is performed in patients with atrial fibrillation (AF) intolerant to long-term anticoagulation [1]. High variability in the incidence of bleeding events after LAAC, ranging from 2.2% to 10% per year across the literature, calls for a more in-depth analysis of post-LAAC bleeding predictors, which in turn could inform a more precise, patient-specific risk assessment and postprocedural treatment regimen choice [2,3,4]. In the current high-volume series, most LAAC recipients have a history of prior bleeding complications in up to 72% [2]. A prior study identified gastrointestinal bleeding history as a predictor of bleeding events after LAAC, suggesting differences in the bleeding rates depending on the susceptible site [5]. Other suggested predictors included age ≥ 75 years [5] and longer postprocedural dual antiplatelet therapy (DAPT) [6]. However, the available data remain limited. Moreover, although procedural bleeding rate might affect overall bleeding outcomes after LAAC, little is known about its short- and long-term consequences [7]. We aimed to study the incidence and predictors of clinically significant bleeding (CSB) after LAAC, particularly bleeding history before the procedure. 

## 2. Materials and Methods

### 2.1. Study Population

This study was an analysis of the single-center registry of all consecutive patients with non-valvular AF who underwent LAAC with an Amplatzer (Abbott, Plymouth, MN, USA) or WATCHMAN (Boston Scientific, Marlborough, MA, USA) device between May 2014 and November 2019 in a single tertiary hospital. All patients signed informed consent before the procedure, and the registry has been approved by the institutional review board.

### 2.2. LAAC Procedure

LAAC was performed in general anesthesia with transesophageal echocardiography (TEE) guidance. Periprocedural medications included: a preprocedural loading dose of acetylsalicylic acid; an intraprocedural dose of unfractionated heparin (with targeted activated clotting time of >250 s); a postprocedural subcutaneous low-molecular-weight heparin (three doses at 12 h intervals starting 4 to 6 h after LAAC) and a loading dose of clopidogrel (the day following the procedure). 

### 2.3. Post-Procedural Antithrombotic Treatment and Follow-Up Assessment

DAPT consisting of aspirin and clopidogrel was the standard therapy after LAAC in the studied cohort. DAPT duration was decided by the treating physician, at the follow-up visits, with the standard protocol of 1 to 6 months of therapy. This was followed by a single antiplatelet therapy (SAPT) or no antiplatelet treatment at all based on the attending physician’s discretion and the presence of alternative indications for antiplatelet therapy than LAAC. Device-related thrombus (DRT), found on follow-up imaging, was managed with a transient anticoagulation introduction or a prolonged dual-antiplatelet therapy and/or observation. Structured follow-up was carried out at a target of 1.5, 3 to 6, and 12 months post-procedure with the planned left atrial imaging at each follow-up visit with TEE or computed tomography (CT). The choice to perform TEE or CT was at the attending physician’s discretion with a preference for TEE in case of reduced estimated glomerular filtration rate.

### 2.4. Study Endpoints and Definition

The main study endpoint was CSB, which was a composite of major (mCRB) and nonmajor (nmCRB) clinically relevant bleeding according to the International Society on Thrombosis and Hemostasis criteria [7,8]. mCRB was defined as fatal or clinically overt bleeding associated with at least one of the following: ≥2 units of blood transfusion, ≥2 g/dL hemoglobin decrease, or a critical anatomic site (intracranial, intraspinal, intraocular, pericardial, intramuscular with compartment syndrome, or retroperitoneal bleeding) [7,8,9]. nmCRB was defined as requiring hospitalization or an invasive procedure but not meeting the above criteria [7,8,9]. CSB was classified as procedure-related (i.e., access-site bleeding or tamponade) and nonprocedural. Nonprocedural CSB was further classified based on the time of its occurrence after LAAC: early (0–1 months), midterm (1–6 months), and late (beyond 6 months). 

Additionally analyzed was an association between procedure-related CSB and hospital length of stay after the procedure, as well as long-term mortality. Cardiovascular death was defined as death attributable to myocardial ischemia, heart failure, cerebrovascular accident or sudden cardiac arrest due to an unknown cause. Noncardiovascular death was defined as all other causes of death. Anemia was defined as a hemoglobin level of less than 13 g/dL in men and less than 12 g/dL in women [10].

### 2.5. Statistical Methods

Continuous variables were presented as median with interquartile range (IQR) and compared using Mann–Whitney test or Kruskal–Wallis analysis of variance (since the hypothesis of normal distribution was rejected in all tested variables in the Shapiro–Wilk test). Categorical variables were presented as frequencies and percentages and compared using Fisher’s exact test. Time-to-event variables were presented as Kaplan–Meier curves. Annualized (per patient-year of follow-up) CSB rate during the post-procedural DAPT period (per patient-year on DAPT) was compared to that after DAPT discontinuation (per patient-year without DAPT). The bleeding rate reduction was assessed by comparing the actual annualized mCRB rate to the annual rate predicted by the HAS-BLED score [9]. The optimal cutoff points for continuous variables predictive of CSB were selected with receiver operating characteristic (ROC) curve analysis according to the maximum Youden Index value. Predictors of CSB were identified using Cox regression with a calculation of hazard ratios (HRs) and a 95% confidence interval (CI). Variables with univariate *p*-values < 0.2 were entered into a multivariable Cox regression with stepwise selection (backward elimination). A *p*-value of <0.05 was considered statistically significant. All statistical analysis was performed using SPSS software, version 25 (SPSS, Inc, Chicago, IL, USA).

## 3. Results

### 3.1. Study Population

A total of 195 consecutive patients (age 74 (IQR, 68–80), 43.1% females, HAS-BLED score of 2.0 (IQR, 2.0–3.0), CHA_2_DS_2_-VASc score of 4.0 (IQR, 3.0–5.0)) with nonvalvular AF who underwent LAAC with WATCHMAN (44.1%) or Amplatzer (55.9%) devices between May 2014 and November 2019 were included in the analysis. The median size of the implanted device was 25 (IQR: 24–28) mm. The median follow-up length was 370 (IQR, 358–392) days; 13 patients (6.7%) died during follow-up and 8 patients (4.1%) were lost to follow-up before the follow-up visit at a target of 12 months. The cause of death was cardiovascular in eight (61.5%) patients, noncardiovascular in three (23.1%) cases (in one case due to massive bleeding), and unknown in two cases (15.4%). The median number of follow-up imaging studies was three (IQR: 2–3) with a 79.0% TEE usage and 11.3% (22/195) incidence of DRT (in 15 out of 22 (68.2%) cases first diagnosed with TEE). The majority of patients were discharged on DAPT, apart from 3.1% (*n* = 6), who were discharged on SAPT due to very high bleeding risk or acetylsalicylic acid intolerance. The median duration of postprocedural DAPT was 91 (IQR: 54–182) days. During follow-up, anticoagulation was temporarily introduced for a median of 78 (IQR: 35–142) days: in nine patients due to the DRT (in five cases (55.6%) non-vitamin K antagonist oral anticoagulant and in four cases (44.4%) low-molecular-weight heparin), whereas in six patients due to other causes (Table S1). 

### 3.2. Bleeding History before LAAC

In the study population, 76.9% of patients (*n* = 150) had pre-LAAC bleeding history (68.2% (*n* = 133) from single anatomical site and 8.7% (*n* = 17) from multiple sites) and 23.1% of patients (*n* = 45) had no pre-LAAC bleeding history. Overall, 30.8% of patients (*n* = 60) had history of gastrointestinal bleeding, 19.0% (*n* = 37) intracranial bleeding, 13.8% (*n* = 27) skin/oral/ocular bleeding, 12.3% (*n* = 24) genitourinary bleeding, and 10.3% (*n* = 20) epistaxis. A comparison of baseline patient characteristics based on pre-LAAC bleeding history is presented in Table S2. Patients with gastrointestinal bleeding history presented the most frequently with estimated glomerular filtration rate (eGFR) < 60 mL/min/1.73 m^2^ (65.4%), anemia (57.7%), and older age than patients without bleeding history. Patients with epistaxis history had the highest rate of coronary artery disease (76.9%), smoking history (53.8%), and reduced left ventricular ejection fraction < 40% (30.8%).

### 3.3. Procedural Outcomes

Procedure-related CSBs (*n* = 17, 8.7%) were vascular access-related complications (*n* = 14, 7.2%) or pericardial bleeding with tamponade (*n* = 3, 1.5%). These were associated with longer hospital length of stay after the procedure (5.0 (IQR: 2.5–9.0) days vs. 2 (IQR: 2.0–3.0) days, respectively, *p* < 0.001) but neither long-term mortality (HR = 0.882, 0.11–6.79, *p* = 0.904) nor prior bleedings history (70.6% (*n* = 12) vs. 77.5% (*n* = 138), respectively, *p* = 0.549) nor future nonprocedural CSB (0.0% (*n* = 0) vs. 7.9% (*n* = 14), respectively, *p* = 0.616). Other non-bleeding procedure-related complications (<7 days post-procedure) included: periprocedural ischemic stroke (*n* = 1), infective endocarditis (*n* = 1), resuscitated cardiac arrest (*n* = 1). 

### 3.4. Nonprocedural Bleeding

**Figure 1:**
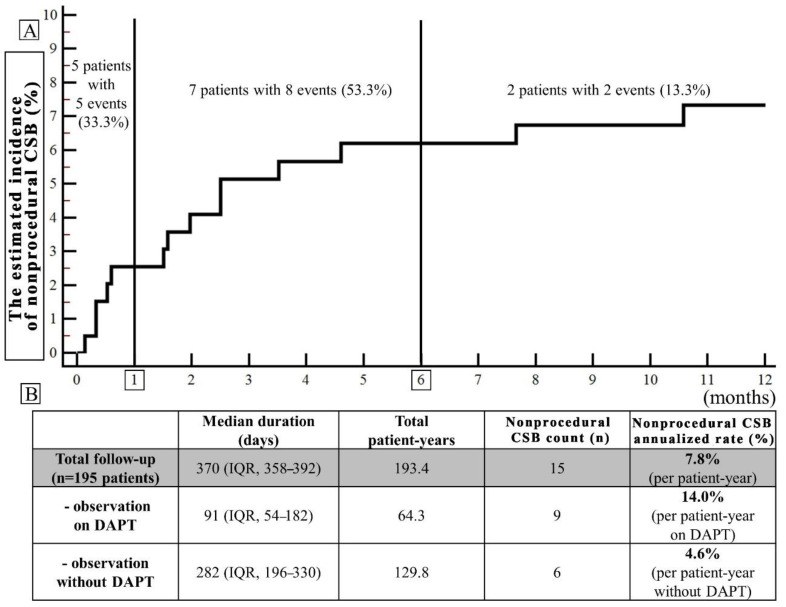
(**A**) The Kaplan–Meier time-to-event curve (the estimated cumulative incidence of the event during the follow-up) for the nonprocedural clinically significant bleedings (CSBs) (major and non-major): early (0–1 month after LAAC), midterm (1–6 months), and late (beyond 6 months). (**B**) Annualized CSB rates (the number of events divided by patient-year of observation expressed as a percentage) separately in the period of observation on dual antiplatelet therapy (DAPT) and without DAPT.

Overall, during the follow-up, there were 15 nonprocedural CSBs in 14 (7.2%) patients corresponding to an annualized rate of 7.8%. In 53.3% (8/15), these were nonprocedural mCRB (Table 1). Median time from LAAC to nonprocedural CSB was 60 (IQR: 17–92) days and nonprocedural CSBs were classified as early in five cases (33.3%), midterm in eight (53.3%) and late in two (13.3%) (Figure 1, Panel A). Overall, nine nonprocedural CSB (60.0%) occurred during postprocedural DAPT (median 46 (IQR: 16–60) days post-LAAC) vs. six (40%) after DAPT discontinuation (median 124 (81–210) days post-LAAC), translating into annualized CBS rates of 14.0% (per patient-year on DAPT) vs. 4.6% (per patient-year without DAPT). In all bleeding cases, DAPT was eventually discontinued during the follow-up period and no CSB was observed thereafter in eight out of nine cases (88.9%). Among the nonprocedural CSB which occurred without DAPT, four out of six (66.7%) occurred during SAPT. None of the CSB occurred during anticoagulation introduced temporarily. 

**Figure 2:**
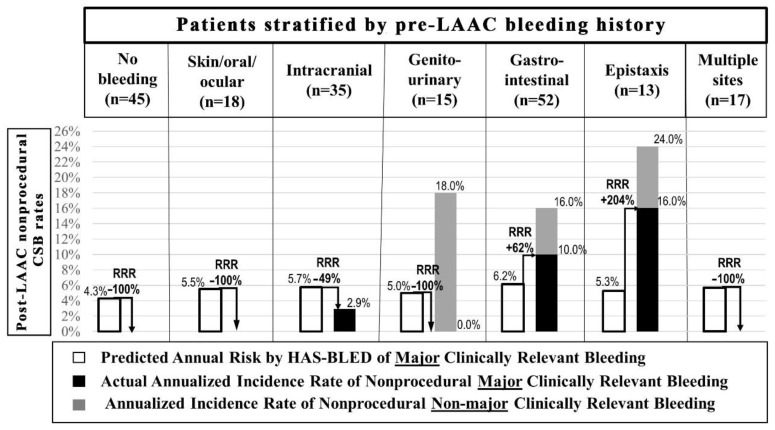
The comparison of annual rates of nonprocedural clinically significant bleedings (a composite of major and non-major clinically relevant bleedings) in patients stratified by pre-LAAC bleeding history. Rates of major clinically relevant bleeding were compared with those predicted by the HAS-BLED score. In 14 out of 15 cases, bleedings were recurrences from the same site as pre-LAAC apart from one patient with pre-LAAC intracranial bleeding history who experienced gastrointestinal bleeding post-LAAC. LAAC = left atrial appendage closure, RRR = relative risk ratio.

Post-LAAC nonprocedural CSB included gastrointestinal bleedings (*n* = 9) (in the majority accompanied by the history of the structural gastrointestinal disorder), epistaxis (*n* = 3) (in two out of three cases associated with the history of hereditary hemorrhagic telangiectasia (HHT)), and genitourinary bleedings (*n* = 3) (Table 1). In 92.9% (13/14) of patients, these were bleeding recurrences from the same site as pre-LAAC (Table 1). A comparison of annual rates of nonprocedural CSB (mCRB and nmCRB) based on bleeding history before LAAC along with mCRB bleeding rates compared to those predicted by the HAS-BLED score is presented in Figure 2. 

Patients with post-LAAC nonprocedural CSB compared to those without such bleeding had higher admission systolic blood pressure (SBP) (140 (131–153) vs. 128 (111–147) mmHg, *p* = 0.026), more frequently pre-LAAC bleeding history (100% vs. 75.1%, *p* = 0.043), permanent AF (78.6% vs. 47.0%, *p* = 0.027), and anemia (64.3% vs. 35.9%, *p* = 0.046) (Table 2). Other baseline characteristics including the HAS-BLED score (2.0 (1.0–3.0) vs. 3.0 (2.0–3.0), *p* = 0.201) were similar (Table 2). Per ROC analysis, admission SBP > 127 mmHg was the optimal cutoff to predict nonprocedural CSB (AUC = 0.678, sensitivity = 93%, specificity = 46%, *p* = 0.005). In the multivariable model, admission SBP > 127 mmHg (HR = 10.73, 1.37–84.26, *p* = 0.024), epistaxis history (HR = 5.84, 1.32–25.89, *p* = 0.020), permanent AF (HR = 4.55, 1.20–17.20, *p* = 0.025), and prior gastrointestinal bleeding (HR = 3.35, 1.01–11.08, *p* = 0.048) were independent predictors of nonprocedural CSB after LAAC (Table 3). 

## 4. Discussion

The main study findings are: (I) nonprocedural CSBs post-LAAC were observed mainly during DAPT (60.0%), in early or midterm post-procedural period (86.6%) (at median 60 (IQR: 17–92) days post-LAAC)) with annualized CSB event rate of 14.0% during postprocedural DAPT (per patient-year on DAPT) vs. 4.6% after DAPT discontinuation (per patient-year without DAPT); (II) the bleeding site in the majority of patients with post-LAAC nonprocedural CSB was the same as before the procedure (92.9%); (III) admission SBP > 127 mmHg, epistaxis history, permanent AF, and prior gastrointestinal bleeding were independent predictors of post-LAAC nonprocedural CSB; (IV) procedure-related CSB increased time to hospital discharge but was not associated with a higher risk of nonprocedural CSB or long-term mortality.

The study identifies patients with a high risk of nonprocedural bleeding after LAAC. Firstly, it confirms the elevated nonprocedural bleeding rate with prior gastrointestinal bleeding history [5,11]. In the contemporary real-life LAAC registries, the majority of patients undergoing the procedure had prior bleeding episodes, with a notably high rate of a gastrointestinal bleeding history [2,11]. Generally, these bleedings were associated with a high recurrence rate that can reach 40% within 1 year following the index event [12]. The reason for this might be persistent structural gastrointestinal disorder predisposing to bleeding. In the LAAC cohort, patients with gastrointestinal bleeding history have high comorbidities burden and advanced age [5]. Gastrointestinal bleeding on anticoagulation was associated with a 13-fold higher hazard of new cancer diagnosis, much higher than with other bleeding types [13]. In line with prior studies in the general population, gastrointestinal bleeding history in this study was associated with impaired estimated glomerular filtration rate, which was also associated with an increased risk of gastritis, peptic ulcer disease, angiodysplasia, and gastrointestinal bleeding [14,15,16]. Secondly, the study documents the novel finding of high bleeding risk in patients with prior epistaxis history. Importantly, two out of three nonprocedural nasal CSB in the follow-up were associated with hereditary hemorrhagic telangiectasia (HHT). HHT patients are prone to develop AF [17] and up to 70% experience heavy bleeding during middle age, even without anticoagulation use [18]. Additionally, the study shows that the LAAC population with epistaxis history poses distinct characteristics with a high burden of overt cardiovascular disease (coronary artery disease) and its risk factors (smoking), which might hypothetically be associated with atherosclerotic changes in nasal arteries promoting bleeding [19,20]. On the other hand, considering the association with reduced ejection fraction seen in the study, increased venous pressure in the nasal vessels, over the course of chronic heart failure, might also be involved [19,20]. Interestingly, some authors proposed a potential explanation for the association between heart failure and recurrent epistaxis of undiagnosed HHT [20]. Thirdly, the nonprocedural bleeding rate was increased in patients with a permanent AF history, which might be due to an increasing rate of comorbidities associated with more sustained AF [21], similarly to the previously described association with increased age ≥ 75 years [5] which, on the other hand, was not confirmed in our study. 

Furthermore, the study suggests important modifiable risk factors that might affect bleeding risk—elevated admission SBP and postprocedural DAPT period. Since prior studies in AF patients suggested the association of SBP with bleeding risk [22,23], documenting a similar relationship in elderly LAAC patients with multiple comorbidities might emphasize the importance of optimization of blood pressure control before the procedure. Our study used routine post-procedural DAPT, and annualized CSB event rate per patient-year on DAPT was 14.0% vs. 4.6% after DAPT discontinuation. Similarly, the EWOLUTION registry suggested the association of longer postprocedural DAPT with major bleedings [6]. Current guidelines suggest DAPT duration ranging from 1 to 6 months [1]. Importantly majority of bleeding events were observed in the mid-term (1–6 months after LAAC) (53.3%), thus in the period of relative indications for DAPT. Ongoing clinical trials might help to establish the optimal DAPT duration [24] and compare the antithrombic regimen with DAPT or non-vitamin K antagonist oral anticoagulants [25]. Interestingly, prior studies in the general population suggested different bleeding rates from various sites in patients with anticoagulant or antiplatelet therapy [26], and some suggested better safety of NOAC than antiplatelet therapy or warfarin in patients with epistaxis [27,28]. 

Despite procedural bleedings remaining the most common of LAAC complications, little is known about their clinical impact. Our study is in line with the observation that these complications might necessitate attention and longer hospital stay [29], but no association with long-term mortality was identified. In addition, the lack of association of procedural bleedings with prior bleeding history and nonprocedural bleedings in the follow-up might suggest a greater role of procedural technique and anatomy rather than patient comorbidities predisposing to procedural bleeding. Thus, meticulous vascular access technique and identifying anatomies predisposing to tamponade [30] might be essential for optimizing outcomes. 

Knowledge about predictors of post-LAAC bleedings, device-related thrombi [31,32] as well as ischemic cerebrovascular events [33] could foster a more personalized treatment approach. The study calls for a more individualized and multidisciplinary approach to bleeding prevention. LAAC in patients with prior bleeding history should probably be accompanied by detailed site-specific diagnostics and treatment since the majority of bleeding events are same-site recurrences. Structural gastrointestinal disorders or vascular malformations might often be involved. Clinical benefits of the LAAC procedure and ways to optimize outcomes should be further studied, especially in the identified high-risk populations. Importantly, hypothetically, the LAAC procedure and discontinuation of anticoagulant treatment in these populations might be especially beneficial because bleeding occurrence on anticoagulation might be associated with a worse prognosis [34]. Whether shortening DAPT and implementing stringent blood pressure control, especially in the presence of high bleeding risk features, may lead to a reduction in bleeding events remains to be studied. 

Due to the low event rate, we do not attempt to analyze the predictive value of DAPT status on bleeding events in time-dependent Cox regression as well as the predictive value of SAPT. We were unable to characterize chronic changes in SBP over time, and we cannot determine from these data whether SBP at admission directly influences outcomes or if it is simply a marker of other processes that influence the outcome.

## 5. Conclusions

CSB after LAAC was related to bleeding history pre-LAAC, in the majority being a recurrence from the same site, with the highest risk among patients with prior gastrointestinal bleeding or epistaxis history during DAPT and significant association with the potentially modifiable risk factor of admission systolic blood pressure.

## Figures and Tables

**Table 1:** Nonprocedural clinically significant bleedings (CSBs) during follow-up per patient.

No.	Post-LAAC CSB Site	Type	Time (Days)	APT	HAS-BLED	Pre-LAAC Bleeding Site	History of Comorbidities Associated with the Pre-LAAC Bleeding Site
1	GI	mCRB	233	single	2	intracranial	-
2	GI	mCRB	10	dual	1	GI	Crohn’s disease, S/P colon resection
3	GI	mCRB	18	dual	3	GI	diverticulosis and polyps in the colon, hemorrhoid disease, S/P chronic gastritis
4	epistaxis	nmCRB	140	none	3	epistaxis	-
5	epistaxis	mCRB	76	dual	1	epistaxis	HHT
6	GI	mCRB	76	dual	2	GI	diverticulosis, S/P polypectomy
7	GI	nmCRB	107	single	1	GI	S/P endoscopic resection of colorectal adenocarcinoma
8	GI	nmCRB	48	dual	1	GI	ulcerative colitis, chronic gastritis
9	epistaxis	mCRB	4	dual	1	epistaxis	HHT
10	GI	nmCRB	10	single	5	GI	gastric ulcer
11	GI	mCRB	46	dual	4	GI	gastric angiodysplasia, S/P argon plasma coagulation
12	genitourinary	nmCRB	16	dual	2	genitourinary	-
13/ 14	genitourinary	nmCRB	60/72	dual/ single	3	genitourinary	-
15	GI	mCRB (fatal)	323	none	2	GI	portal hypertension, alcoholic liver cirrhosis, peptic ulcer disease

APT = antiplatelet therapy during the event; CSB = clinically significant bleeding; GI = gastrointestinal bleeding; HHT = hereditary hemorrhagic telangiectasia; LAAC = left atrial appendage closure; mCRB = major clinically relevant bleeding; nmCRB = non-major clinically relevant bleeding, S/P = status post.

**Table 2:** Comparison of baseline clinical, echocardiographic, and procedural characteristics based on the occurrence of nonprocedural clinically significant bleeding during follow-up.

Variable	CSB after LAAC (*n* = 14)	No CSB after LAAC (*n* = 181)	*p*-Value
Female gender, *n* (%)	5 (35.7)	79 (43.6)	0.780
Age (years); median (IQR)	69 (56–81)	74 (68–80)	0.240
Age ≥ 75 years	6 (42.9)	88 (48.6)	0.785
HAS-BLED score; median (IQR)	2.0 (1.0–3.0)	3.0 (2.0–3.0)	0.201
Predicted annual bleeding risk; median (IQR)	4.1 (3.4–5.8)	5.8 (4.1–5.8)	0.203
Comorbidities			
Hypertension, *n* (%)	12 (85.7)	154 (85.1)	1.000
Diabetes mellitus, *n* (%)	4 (28.6)	55 (30.4)	1.000
Smoking history, *n* (%)	5 (35.7)	47 (26.0)	0.530
Prior ischemic stroke/TIA/PE, *n* (%)	3 (21.4)	57 (31.5)	0.556
Coronary artery disease, *n* (%)	5 (35.7)	85 (47.8)	0.420
PCI/CABG history, *n* (%)	3 (21.4)	69 (38.1)	0.261
MI history, *n* (%)	2 (14.3)	53 (29.3)	0.357
Carotid/peripheral artery disease, *n* (%)	1 (7.1)	31 (17.1)	0.473
Pacemaker/ICD implanted, *n* (%)	3 (21.4)	42 (23.2)	1.000
Permanent atrial fibrillation, *n* (%)	11 (78.6)	85 (47.0)	0.027
Cancer history, *n* (%)	1 (7.1)	29 (16.0)	0.700
Venous thromboembolism history, *n* (%)	0 (0.0)	12 (6.6)	1.000
Bleeding history			
Bleedings history, *n* (%)	14 (100)	136 (75.1)	0.043
Skin/oral/ocular bleeding, *n* (%)	0 (0.0)	27 (14.9)	0.224
Intracranial bleeding, *n* (%)	1 (7.1)	36 (19.9)	0.476
Genitourinary bleeding, *n* (%)	2 (14.3)	22 (12.2)	0.684
Gastrointestinal bleeding, *n* (%)	8 (57.1)	52 (28.7)	0.036
Epistaxis, *n* (%)	3 (21.4)	17 (9.4)	0.161
Bleedings history while on anticoagulation, *n* (%)	11 (78.6)	113 (62.4)	0.265
Transthoracic ECHO parameters			
LVEF < 40%, *n* (%)	1 (7.1)	14 (7.7)	1.000
Moderate-to-severe mitral regurgitation, *n* (%)	1 (7.1)	29 (16.0)	0.700
Laboratory results			
Hemoglobin (g/dL); median (IQR)	12.2 (11.0–13.2)	13.1 (11.8–14.6)	0.071
Anemia, *n* (%)	9 (64.3)	65 (35.9)	0.046
Platelets (10^3^/mL); median (IQR)	192 (172–226)	183 (139–231)	0.419
eGFR < 60 mL/min/1.73 m^2^, *n* (%)	4 (28.6)	89 (49.2)	0.170
Admission SBP (mmHg); median (IQR)	140 (131–161)	130 (114–148)	0.026
Admission DBP (mmHg); median (IQR)	80 (77–82)	76 (67–85)	0.131
Admission heart rate (beats/min); median (IQR)	75 (70–83)	73 (64–83)	0.283

CABG = coronary artery bypass grafting; DBP = diastolic blood pressure; eGFR = estimated glomerular filtration rate; ICD = implantable cardioverter–defibrillator; IQR = interquartile range; LAAC = left atrial appendage closure; LVEF = left ventricle ejection fraction; MI = myocardial infarction; PCI = percutaneous coronary intervention; PE = peripheral embolus; SBP = systolic blood pressure; TIA = transient ischemic attack.

**Table 3:** Predictors of nonprocedural clinically significant bleeding. AF = atrial fibrillation; eGFR = estimated glomerular filtration rate; SBP = systolic blood pressure.

	Univariate		Multivariable		
	HR (95%CI)	*p*-Value	HR (95%CI)		*p*-Value
Admission SBP > 127 mmHg	10.38 (1.36–79.37)	0.024	10.73 (1.37–84.26)		0.024
Epistaxis history	2.45 (0.68–8.76)	0.170	5.84 (1.32–25.89)		0.020
Permanent AF	3.90 (1.09–13.97)	0.037	4.55 (1.20–17.20)		0.025
Gastrointestinal bleeding history	3.23 (1.12–9.31)	0.030	3.35 (1.01–11.08)		0.048
Anemia	3.15 (1.06–9.40)	0.040			
eGFR <60 mL/min/1.73 m^2^	0.45 (0.14–1.42)	0.172			

## Data Availability

Not applicable.

## Supplementary Materials

The following supporting information can be downloaded at: https://www.mdpi.com/article/10.3390/ijerph192113802/s1, Table S1. Indications for temporary reintroduction of anticoagulant treatment. Table S2. Comparison of baseline clinical, echocardiographic and procedural characteristics stratified according to bleeding history before LAAC.
