# Supplementary material for: Incidence and Predictors of Clinically Significant Bleedings after Transcatheter Left Atrial Appendage Closure
Source: Int J Environ Res Public Health. 2022 Oct 24;19(21):13802. doi: 10.3390/ijerph192113802 (PMC9658798; doi:10.3390/ijerph192113802)
Supplement: Supplementary file 1 [file ijerph-19-13802-s001.zip › ijerph-1919744-supplementary.pdf]

Table S1. Indications for temporary reintroduction of anticoagulant treatment.

Indications	Number of patients
Device-related thrombus	9
Significant peridevice leak	2
Native mitral valve thrombus	1
Superficial thrombophlebitis	1
Peripheral artery disease	1
Temporary alternative to dual antiplatelet therapy	1

Table S2. Comparison of baseline clinical, echocardiographic and procedural characteristics stratified according to bleeding history before LAAC.

Variable	No bleeding history (*n*=45)	Skin/oral/ocular bleeding (*n*=18)	Intracranial bleeding (*n*=35)	Genitourinary bleeding (*n*=15)	Gastrointestinal bleeding (*n*=52)	Epistaxis (*n*=13)	Multiple sites (*n*=17)	*p*-value
Female gender, *n* (%)	22 (48.9)	11 (61.1)	13 (37.1)	6 (40.0)	23 (44.2)	2 (15.4)	7 (41.2)	0.260
Age (years); median (IQR)	73 (61–79)	77 (69–84)*	73 (66–79)	73 (64–80)	77 (69–83)*	74 (65–84)	76 (69–82)	0.191
HAS-BLED score; median (IQR)	2 (1–3)	2 (1–4)	3 (2–4)*	2 (2–3)	3 (2–4)*	2 (2–3)	3 (2–4)*	0.004
Predicted annual bleeding risk; median (IQR)	4 (3–6)	4 (3–9)	6 (4–9)*	4 (4–6)	6 (4–9)*	4 (4–6)	6 (4–7)*	0.004
Comorbidities								
Hypertension, *n* (%)	38 (84.4)	16 (88.9)	31 (88.6)	12 (80.0)	44 (84.6)	11 (84.6)	14 (82.4)	0.982
Diabetes mellitus, *n* (%)	15 (33.3)	4 (22.2)	15 (42.9)	5 (33.3)	11 (21.2)	4 (30.8)	5 (29.4)	0.479
Smoking history, *n* (%)	10 (22.2)	3 (16.7)	13 (37.1)	3 (20.0)	11 (21.2)	7 (53.8)*	5 (29.4)	0.180
Prior ischemic stroke/TIA/PE, *n* (%)	28 (62.2)	3 (16.7)*	6 (17.1)*	4 (26.7)*	13 (25.0)*	3 (23.1)*	3 (17.6)*	<0.001
Coronary artery disease, *n* (%)	16 (35.6)	11 (64.7)	12 (34.3)	5 (35.7)	28 (54.9)	10 (76.9)*	8 (47.1)	0.037
PCI/CABG history, *n* (%)	13 (28.9)	9 (50.0)	9 (25.7)	4 (26.7)	22 (42.3)	9 (69.2)*	6 (35.3)	0.079
MI history, *n* (%)	8 (17.8)	6 (33.3)	8 (22.9)	2 (13.3)	17 (32.7)	6 (46.2)	8 (47.1)*	0.110
Carotid/peripheral artery disease, *n* (%)	8 (17.8)	3 (16.7)	5 (14.3)	2 (13.3)	8 (15.4)	1 (7.7)	5 (29.4)	0.836
Pacemaker/ICD implanted, *n* (%)	9 (20.0)	6 (33.3)	7 (20.0)	5 (33.3)	10 (19.2)	4 (30.8)	4 (23.5)	0.737
Permanent atrial fibrillation, *n* (%)	19 (42.2)	5 (27.8)	16 (45.7)	9 (60.0)	29 (55.8)	9 (69.2)	9 (52.9)	0.235
Cancer history, *n* (%)	4 (8.9)	3 (16.7)	3 (8.6)	4 (26.7)	11 (21.2)	3 (23.1)	2 (11.8)	0.349
Venous thromboembolism history, *n* (%)	2 (4.4)	1 (5.6)	1 (2.9)	0 (0.0)	3 (5.8)	3 (15.4)	3 (17.6)	0.263
Bleedings history while on anticoagulation, *n* (%)	0 (0.0)	13 (72.2)*	28 (80.0)*	15 (100)*	42 (80.8)*	12 (92.3)*	14 (82.4)*	<0.001

Transthoracic ECHO parameters								
LVEF <40%, *n* (%)	1 (2.2)	3 (16.7)	3 (8.6)	0 (0.0)	3 (5.8)	4 (30.8)*	1 (5.9)	0.030
Moderate-to-severe mitral regurgitation, *n* (%)	5 (11.1)	2 (11.1)	6 (17.1)	1 (6.7)	14 (26.9)	0 (0.0)	2 (11.8)	0.138
Laboratory results								
Hemoglobin (g/dL); median (IQR)	13 (12–15)	13 (11–15)	14 (12–15)	14 (13–15)	12 (11–13)*	13 (11–15)	13 (11–14)	0.007
Anemia, *n* (%)	13 (28.9)	4 (22.2)	7 (20.0)	3 (20.0)	30 (57.7)*	8 (61.5)	9 (52.9)	0.001
Platelets (10^3/mL); median (IQR)	183 (135–225)	205 (137–244)	183 (152–226)	183 (150–220)	181 (136–235)	184 (123–233)	183 (122–228)	0.994
eGFR <60 ml/min/1.73m^2, *n* (%)	18 (40.0)	10 (55.6)	10 (28.6)	6 (40.0)	34 (65.4)*	6 (46.2)	9 (52.9)	0.030

*p<0.05 for the difference in comparison to the group with no bleeding history.

CABG = coronary artery bypass grafting; eGFR = estimated glomerular filtration rate; ICD = implantable cardioverter–defibrillator; IQR = interquartile range; LAAC = left atrial appendage closure; LVEF = left ventricle ejection fraction; MI = myocardial infarction; PCI = percutaneous coronary intervention; PE = peripheral embolus; TIA = transient ischemic attack
